# Average firing rate rather than temporal pattern determines metabolic cost of activity in thalamocortical relay neurons

**DOI:** 10.1038/s41598-019-43460-8

**Published:** 2019-05-06

**Authors:** Guosheng Yi, Warren M. Grill

**Affiliations:** 1 Department of Biomedical Engineering, Duke University, Durham, NC United States; 2 Department of Electrical and Computer Engineering, Duke University, Durham, NC United States; 3 Department of Neurobiology, Duke University School of Medicine, Durham, NC United States; 4 Department of Neurosurgery, Duke University School of Medicine, Durham, NC United States; 5 School of Electrical and Information Engineering, Tianjin University, Tianjin, China

**Keywords:** Computational models, Biophysical models

## Abstract

Thalamocortical (TC) relay cells exhibit different temporal patterns of activity, including tonic mode and burst mode, to transmit sensory information to the cortex. Our aim was to quantify the metabolic cost of different temporal patterns of neural activity across a range of average firing rates. We used a biophysically-realistic model of a TC relay neuron to simulate tonic and burst patterns of firing. We calculated the metabolic cost by converting the calculated ion fluxes into the demand for ATP to maintain homeostasis of intracellular ion concentrations. Most energy was expended on reversing Na^+^ entry during action potentials and pumping Ca^2+^ out of the cell. Average firing rate determined the ATP cost across firing patterns by controlling the overall number of spikes. Varying intraburst frequency or spike number in each burst influenced the metabolic cost by altering the interactions of inward and outward currents on multiple timescales, but temporal pattern contributed substantially less to the metabolic demand of neural activity as compared to average firing rate. These predictions should be considered when interpreting findings of functional imaging studies that rely of estimates of neuronal metabolic demand, e.g., functional magnetic resonance imaging.

## Introduction

Thalamocortical (TC) relay neurons exhibit two distinct firing patterns - tonic and burst^1–3^ - that determine their manner of information processing and transmission of signals to the cortex^1,4^. Differences in metabolic demand between these firing modes are critical to interpreting functional brain imaging methods that infer neural activity from related metabolic mechanisms, for example functional magnetic resonance imaging (fMRI). Specifically, *in vivo* recordings suggest a tight correspondence between blood-oxygen-level dependent (BOLD) fMRI signals and neural activity^5–9^. However, it is not clear how the temporal pattern of neural activity, as opposed to the average firing rate of neurons, contributes to metabolic demand, and therefore might influence measures in functional brain imaging.

Processes consuming metabolic energy include maintaining rest potential, restoring ionic concentration gradients after action potentials (APs), reversing transmitter-evoked ionic fluxes through postsynaptic receptors, neurotransmitter recycling, reversing presynaptic AP-triggered Ca^2+^ entry, and vesicle cycling^10,11^. An energy budget of neural computation showed that most signaling-related energy was expended to pump Na^+^ and Ca^2+^ ions out of cells against their electrochemical gradients^10^. By converting ion fluxes into the number of ATP molecules, the metabolic cost of APs was determined in different cell types and species, and the effects of AP shape^12^, temperature^13^, channel densities and kinetics^14^, cell size^15^, and dendritic nonlinearity^16^ were considered. The metabolic cost of a single AP was dependent on the firing frequency^11,13–16^, and computational models predicted that metabolic cost per spike varied inversely with the firing rate during spike frequency adaptation^16,17^. Indeed, experimental recordings made in lateral superior olive, hippocampal CA1 region, and cerebral cortex indicated that the kinetics of ATP consumption and regeneration of energy equivalents, including nicotinamide adenine dinucleotide, flavin adenine dinucleotide, and O_2_ concentration, were correlated with the frequency of neural activity^18,19^.

The objective of this study was to estimate the metabolic cost of different temporal patterns of neural activity across a range of average firing rates. We used a computational model to simulate tonic and burst patterns of firing in TC relay neurons. Our results revealed that the metabolic demand to restore ionic concentration gradients during neural activity was determined by average firing rate, and the pattern of activity contributed little to the energy cost.

## Methods

### TC relay model

**Figure 1:**
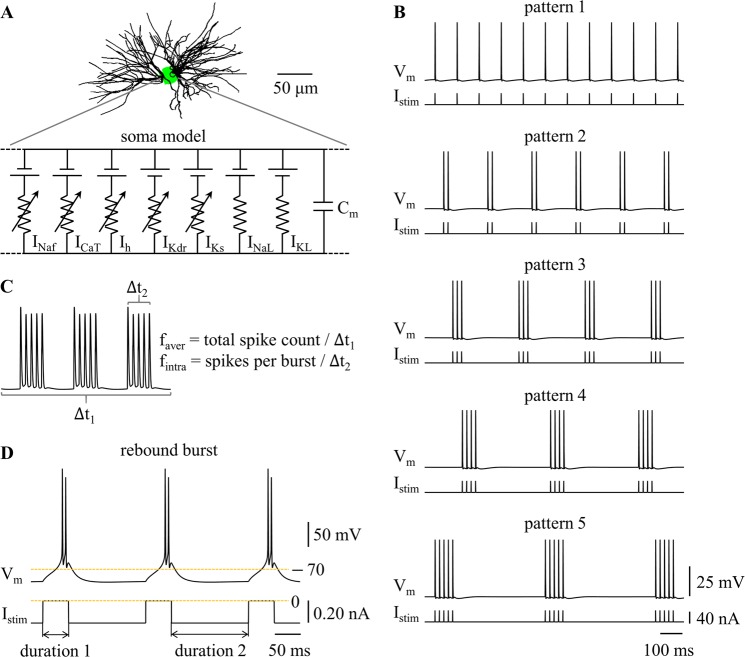
Firing patterns in a computational model of a TC relay neuron. (**A**) Schematic of the model. (**B**) Temporal patterns of neural activity. Transmembrane voltage V_m_ was measured in the cell body. Depolarizing pulse train I_stim_ (pulse width: 0.1 ms, pulse amplitude: 40 nA) was applied to cell body. (**C**) Graphical depiction of *f*_aver_ and *f*_intra_. *f*_aver_ = total spike count/Δt_1_, and *f*_intra_ = spikes per burst/Δt_2_. (**D**) Rebound burst at *f*_aver_ = 10 Hz. Hyperpolarizing current (amplitude: −0.2 nA) was injected in the cell body. Duration 1 was 50 ms, and duration 2 was 150 ms.

We used a computational model to simulate different temporal patterns of neural activity in TC relay neurons. The model included a dendritic tree, a cell body, and a myelinated axon, and its morphology was reconstructed from a filled TC cell from rat ventrobasal nucleus^20^. We examined the metabolic cost of neural activity in the cell body, as due to accessibility, this was the common site of recording in most experiments. There were three compartments in the soma (Fig. 1A), which were modeled with non-linear membrane dynamics including the parallel combination of fast Na^+^ (I_Naf_), delayed rectifier K^+^ (I_Kdr_), T-type Ca^2+^ (I_CaT_), hyperpolarization-activated cation (I_h_), slow K^+^ (I_Ks_), Na^+^ linear leakage (I_NaL_), and K^+^ linear leakage (I_KL_) currents, and membrane capacitance (C_m_). The details of the model followed McIntyre *et al*.^21^, and the model replicated a wide range of electrophysiological properties of TC neurons.

### Calculation of metabolic cost

The metabolic cost of different patterns of neural activity was determined by the number of ATP molecules expended to pump Na^+^ and Ca^2+^ ions out of the cell. Since the Na^+^/K^+^ ATPase pump did not differentiate between Na^+^ channel subtypes, we determined Na^+^ influx by summing I_NaL_ and I_Naf_^10^. Ca^2+^ entry during APs or membrane hyperpolarization occurred through T-type Ca^2+^ channels, and to restore and maintain intracellular Ca^2+^ levels, the Ca^2+^-ATPase or Na^+^-Ca^2+^ exchanger pumped the Ca^2+^ entry out of the cell^11^. Therefore, the calculation of ATP consumption included the Na^+^ influx during APs, the Na^+^ influx at rest, and Ca^2+^ entry.

The total Na^+^ entry was computed by integrating the area under I_Naf_(t) and I_NaL_(t) curves. Since one Ca^2+^ ion has two positive charges, the Ca^2+^ entry was computed by integrating I_CaT_(t) curve, divided by 2. The Na^+^/K^+^ pump hydrolyzes one ATP molecule for every three Na^+^ ions extruded, and the Ca^2+^-ATPase or Na^+^-Ca^2+^ exchanger consumes one ATP for every Ca^2+^ ion extruded^10,11^. Thus, the number of ATP molecules consumed in each compartment was calculated by: ${{\rm{S}}}_{{\rm{com}}}\ast \frac{{{\rm{N}}}_{{\rm{A}}}}{{\rm{F}}}[\frac{{\int }^{}({{\rm{I}}}_{{\rm{Naf}}}+{{\rm{I}}}_{{\rm{NaL}}}){\rm{dt}}}{3}+\frac{\int {{\rm{I}}}_{{\rm{CaT}}}{\rm{dt}}}{2}]$, where S_com_ was the membrane area of the compartment (ionic currents I were current densities), N_A_ was Avagadro’s number, and F was Faraday’s constant. The total metabolic cost was the sum of the ATP consumed in each of the three somatic compartments. This value was then divided by the time duration of the simulation to get a rate of metabolic energy consumed (in mol/s) during different rates and patterns of neural firing.

The resting potential was −70.40 mV in TC model neuron, which was an emergent property dependent upon all conductances and reversal potentials that were represented in the model. Although the net membrane current was 0 mA/cm^2^ at rest, there were still ion fluxes through relevant channels. Specifically, the Na^+^ influx mainly occurred through Na^+^ leakage channels (I_NaL_ = 1.10 × 10^−3^ mA/cm^2^), and the fast Na^+^ current (I_Naf_ = 3.17 × 10^−6^ mA/cm^2^) made little contribution. The Ca^2+^ influx occurred through T-type Ca^2+^ channels (I_CaT_ = 1.65 × 10^−4^ mA/cm^2^). To maintain the resting potential, the Na^+^/K^+^ and Ca^2+^ pumps reversed these ion fluxes to maintain the concentration gradient for each ion, which consumed the ATP at a rate of 8.7770 × 10^7^ mol/s. 81.51% of the ATP was consumed to reverse the Na^+^ leak, 18.31% was to pump the Ca^2+^ ions, and less than 1% was consumed to reverse leakage through the fast Na^+^ channels.

### Simulation

We implemented the TC model in NEURON (v7.5)^22^, and the simulations were run with a time step of 0.025 ms. We quantified the metabolic cost of five temporal patterns of neural activity, which are illustrated in Fig. 1B. Pattern 1 was tonic firing, and patterns 2–5 were burst firing. Each pattern was recorded for 10 periods after a 1000 ms period of initialization. The average firing rate *f*_aver_ and intraburst frequency *f*_intra_ were used to quantify the burst patterns, and *f*_aver_ was used to quantify the tonic patterns. *f*_aver_ referred to the average firing rate during the whole stimulation train, and *f*_intra_ was the firing rate within each burst (Fig. 1C). We used 0 mV as a threshold to detect APs in the cell body.

We applied depolarizing pulse trains I_stim_ to the cell body to generate the five firing patterns. We recorded transmembrane potential and transmembrane currents in the soma. We examined the effects of *f*_aver_ and *f*_intra_ on the metabolic cost of each pattern of firing. Pulse amplitude was 40 nA and pulse width was 0.1 ms, which were suprathreshold for activation of APs in the cell body as *f*_aver_ or *f*_intra_ varied. The current pulses controlled the firing patterns in the cell body while themselves producing little effect on the metabolic cost of neural activity. Thus, this approach allowed us to quantify how the average firing rate and temporal pattern influenced the metabolic cost of neural activity.

Tonic and burst firing in TC relay neurons is typically generated by a large number of synaptic inputs arriving at the dendrites. We conducted additional simulations to quantify the metabolic cost of the five firing patterns generated by synaptic inputs, rather than by current injection into the soma. Both approaches – synaptic inputs distributed across the dendritic tree and somatic current injection – generated identical patterns of neural activity (Supplementary Fig. S1). Further, these patterns generated very similar metabolic costs across changes in rate and pattern (Supplementary Figs S2, S3). Thus, our approach of using somatic current injection to generate and control firing activity did not influence our predictions of the effects of firing pattern on metabolic demand.

De-inactivation of I_CaT_ generates low-threshold Ca^2+^ spikes (i.e., rebound bursts) in TC neurons^1,3^, and also results in burst patterns of firing. During Ca^2+^-mediated spikes, the intraburst frequency *f*_intra_ was dominated by the rebound depolarization, and we only examined the effects of *f*_aver_ on the metabolic cost of neural activity. We applied constant hyperpolarizing current pulses to the cell body^3^ and 50 ms pauses in the hyperpolarizing current (duration 1, i.e., current switched off) resulted in low-threshold rebound spikes (Fig. 1D). The interval between pauses in the hyperpolarizing current (duration 2) changed the average firing rate *f*_aver_, and increasing the amplitude of the hyperpolarizing phase increased the size of rebound depolarization, thereby increasing the number of spikes in each burst. Since the number of spikes in each burst also varied with duration 2, we used the smallest-amplitude of the hyperpolarizing current required to generate firing patterns at each average firing rate.

## Results

### Effects of average firing rate

**Figure 2:**
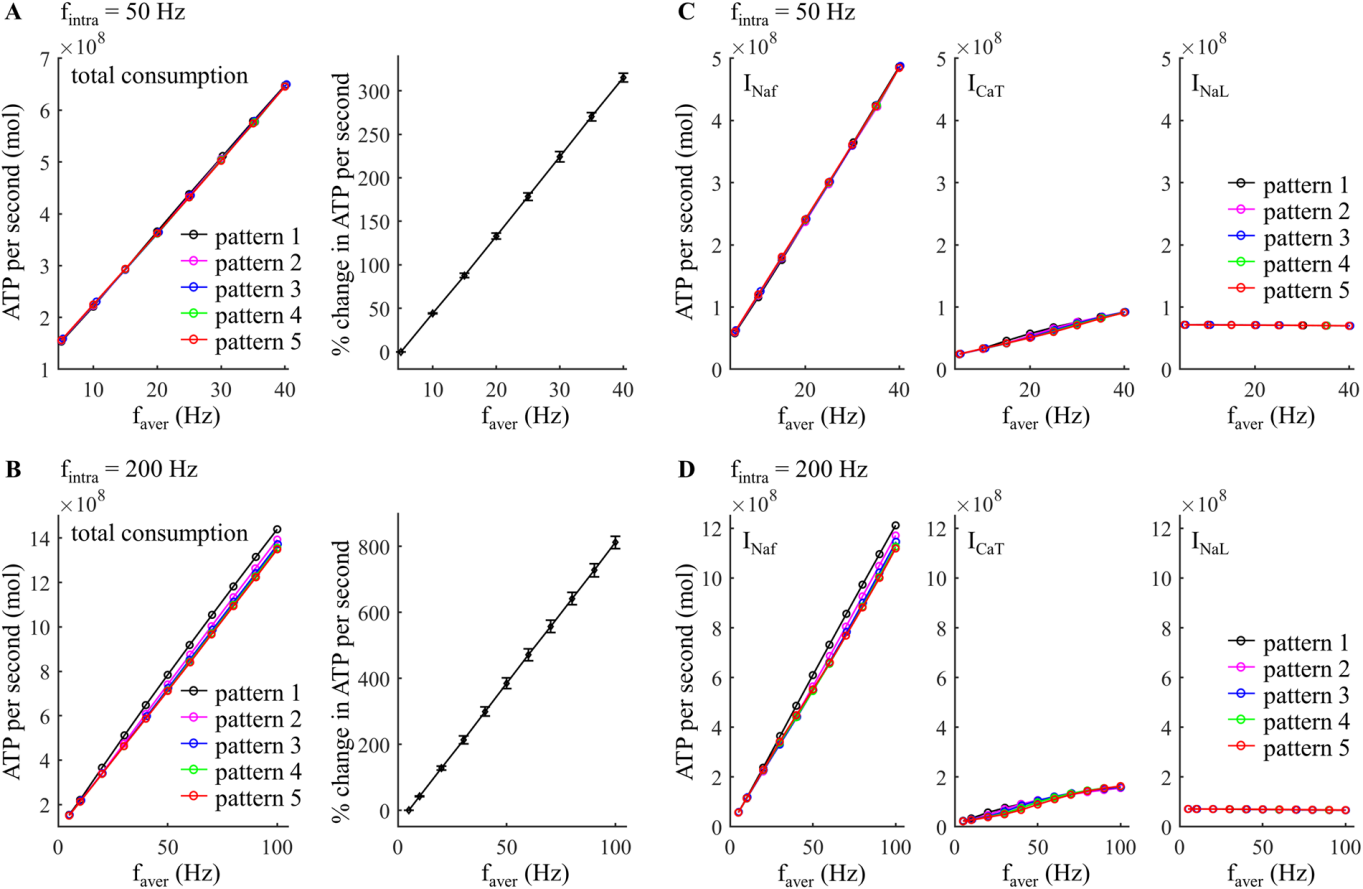
Effect of average firing rate, *f*_aver_, on estimated metabolic cost of different temporal patterns of neural activity. (**A**,**B**) Left: total ATP consumption at *f*_intra_ = 50 Hz and 200 Hz. Right: percent change in total ATP consumption (mean ± SD across five patterns). (**C**,**D**) ATP consumption required to reverse the ionic fluxes through each of I_Naf_, I_CaT_, and I_NaL_ at *f*_intra_ = 50 Hz and 200 Hz.

**Figure 3:**
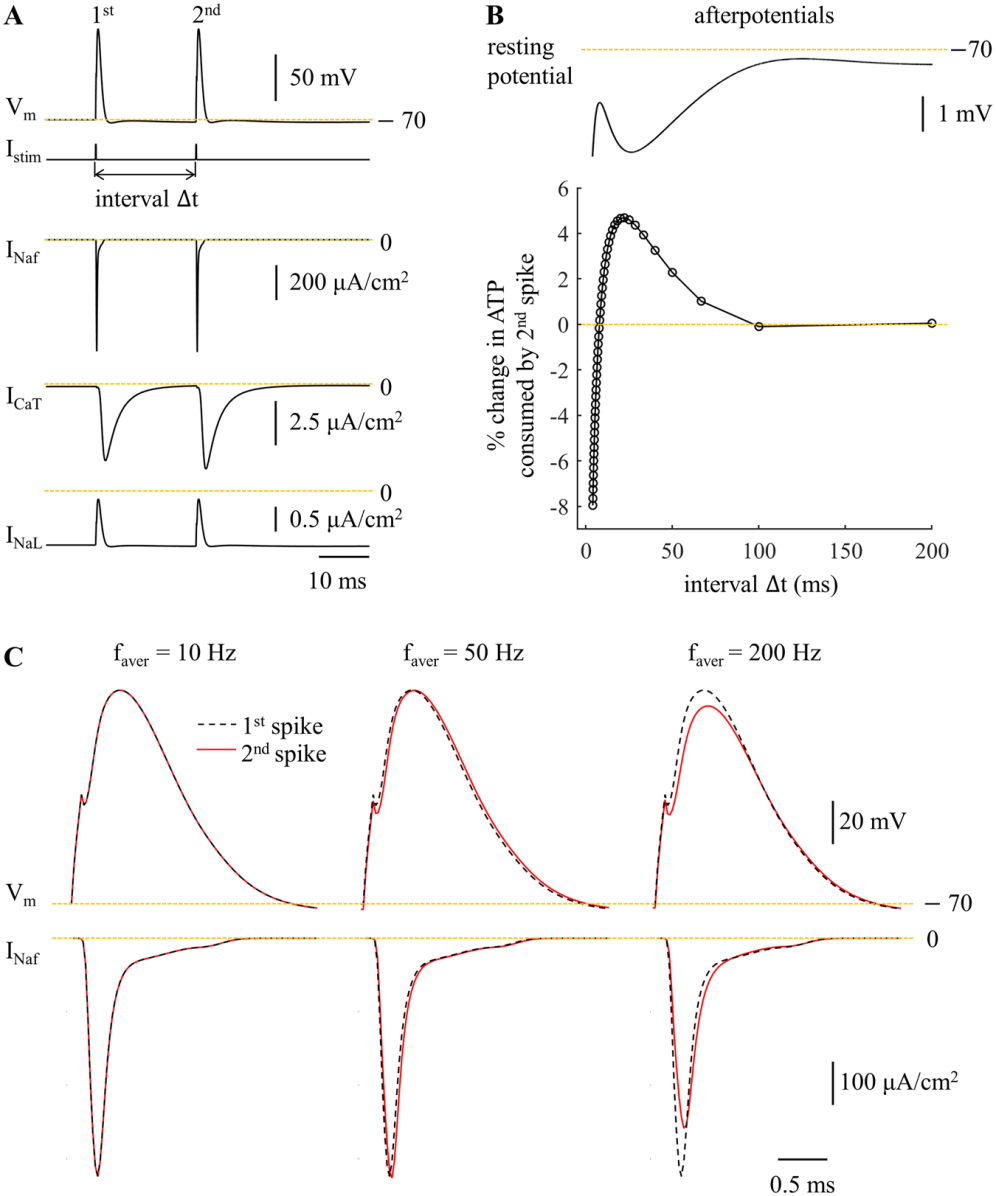
Metabolic cost of pairs of APs as a function of the inter-pulse interval reflect the contributions of afterpotentials. (**A**) Sample responses and underlying currents (including I_Naf_, I_CaT_, and I_NaL_). Negative currents were inward. Two depolarizing pulses (amplitude: 40 nA, width: 0.1 ms) were applied to the cell body, and the inter-pulse interval was Δt = 20  ms. (**B**) Top: afterpotentials on the same time scale as the inter-stimulus interval, Δt. Bottom: percent change in ATP cost for the 2^nd^ AP relative to the 1^st^ AP as a function of Δt. The interval Δt was calculated by 1/*f*_aver_, and *f*_aver_ was from 5 Hz to 250 Hz at a step size of 5 Hz. (**C**) Fast Na^+^ currents underlying two APs at *f*_aver_ = 10 Hz, 50 Hz, and 200 Hz.

We first quantified the effects of average firing rate *f*_aver_ on the metabolic cost of each firing pattern generated by trains of depolarizing pulses injected into the cell body. ATP demand increased linearly as a function of *f*_aver_ and exhibited very similar trends across the five temporal patterns (Fig. 2A,B). The depolarization phase of the APs generated abundant Na^+^ entry through fast Na^+^ channels (Fig. 3A), which dominated the metabolic cost of neural activity. Increasing *f*_aver_ increased the number of spikes in each pattern of firing, and thus increased the ATP consumption to pump out the Na^+^ that entered through the fast Na^+^ channel (Fig. 2C,D, left). Although depolarizing pulses inactivated low-threshold I_CaT_, a small number of Ca^2+^ ions entered through T-type Ca^2+^ channels during each AP (Fig. 3A), and the ATP expended on Ca^2+^ extrusion also increased with *f*_aver_ (Fig. 2C,D, center). In contrast, Na^+^ entry through I_NaL_ was constant during the interspike intervals and was much smaller than I_Naf_ (Fig. 3A). Since increasing numbers of APs reduced I_NaL_, the ATP expended to reverse Na^+^ leak decreased with *f*_aver_ (Fig. 2C,D, right).

At *f*_intra_ = 50 Hz, there was little difference in total ATP consumption between firing patterns (Fig. 2A, left). At *f*_intra_ = 200 Hz, burst patterns consumed slightly less ATP than tonic patterns, and increasing numbers of spikes in each burst slightly reduced the metabolic cost of neural activity in comparison to patterns with fewer spikes per burst (Fig. 2B, left). To understand further the role of pattern on metabolic demand, we applied two suprathreshold pulses (amplitude: 40 nA, width: 0.1 ms) to the cell body to evoke pairs of APs, and the metabolic cost of the 2^nd^ spike was determined as a function of the inter-pulse interval (Fig. 3B). The Na^+^ influx by the 2^nd^ spike was dominated by the voltage-dependent I_Naf_, which exhibited different degrees of activation dependent upon the hyperpolarizing afterpotentials from the 1^st^ spike. At low frequencies (≤10 Hz), the inter-pulse interval was long enough that there was little change in the I_Naf_ underlying the two spikes (Fig. 3C), and thus the metabolic costs were similar. At moderate frequencies (from 15 Hz to 125 Hz), I_Naf_ activated more slowly during the 2^nd^ AP, which resulted in a small delay of the fast Na^+^ current. This increased Na^+^ influx during the 2^nd^ spike, and its ATP demand was higher than the 1^st^ spike. However, the ATP consumed to extrude Ca^2+^ entry was reduced from pattern 1 to pattern 5 (Fig. 2C, center), and the total consumption of neural activity exhibited little difference between firing patterns at *f*_intra_ = 50 Hz. At high frequencies (≥130 Hz), the afterpotentials attenuated the I_Naf_ underlying the 2^nd^ spike, thereby reducing total Na^+^ influx and the ATP cost of burst patterns.

### Effects of intraburst frequency

**Figure 4:**
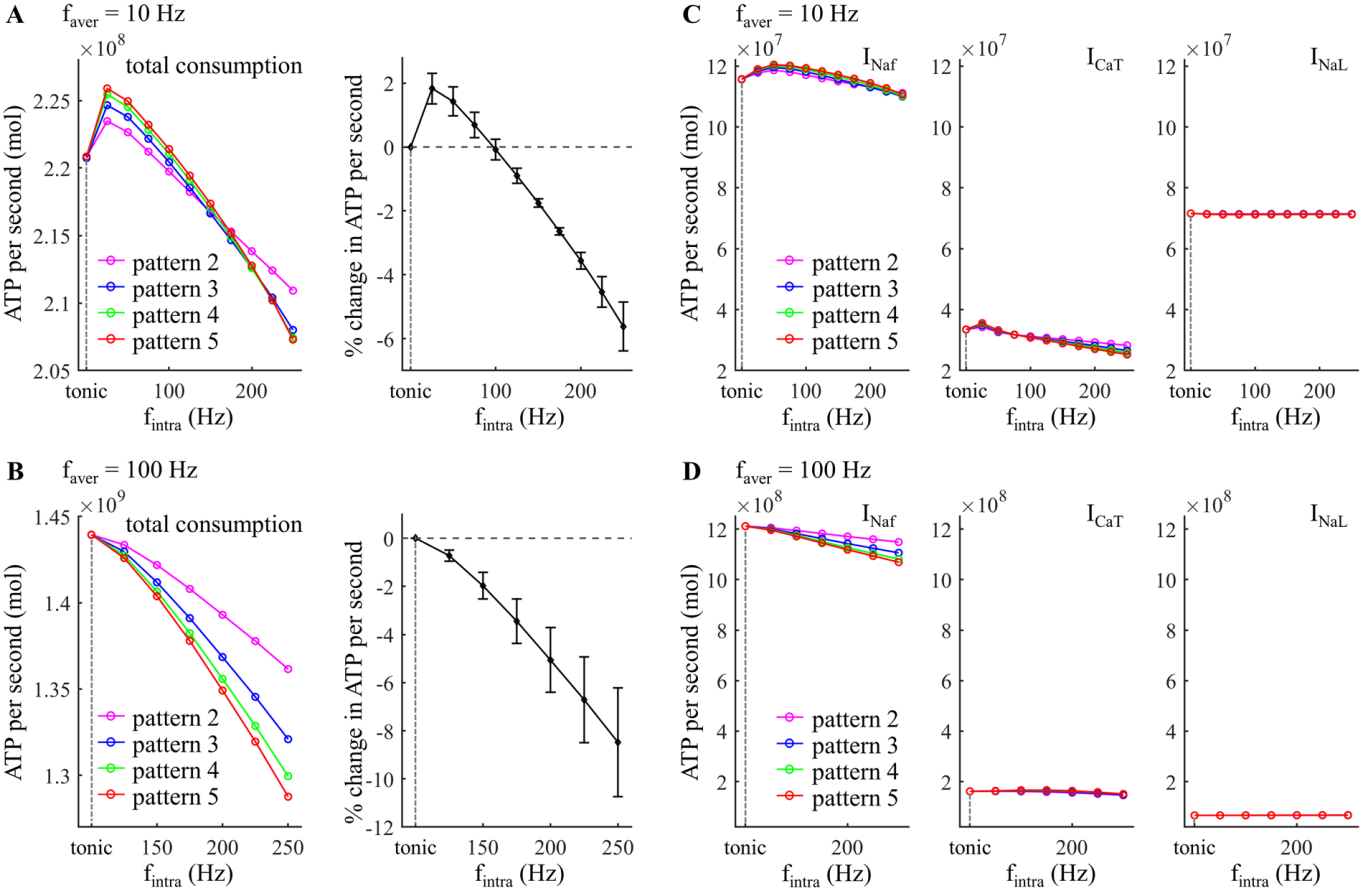
Effect of *f*_intra_ on estimated metabolic cost of different temporal patterns of neural activity. (**A**,**B**) Left: total ATP consumption at *f*_aver_ = 10 Hz and 100 Hz. Right: percent change in total ATP consumption (mean ± SD across five patterns). (**C**,**D**) ATP consumption required to reverse the ionic fluxes through each of I_Naf_, I_CaT_, and I_NaL_ at *f*_aver_ = 10 Hz and 100 Hz.

We quantified the effects of *f*_intra_ on the metabolic cost of five temporal patterns of activity generated by applying depolarizing pulses to the cell body. Compared to varying *f*_aver_, increasing *f*_intra_ produced much smaller effects on the ATP demand. A 100 Hz increment in *f*_intra_ resulted in less than 10% change of the mean ATP cost across firing patterns (Fig. 4A,B, right), but an only 5 Hz increment in *f*_aver_ increased the mean ATP cost by more than 43% (Fig. 2A,B, right). At *f*_aver_ = 10 Hz, the metabolic cost of burst patterns declined slightly as *f*_intra_ increased (Fig. 4A). The increased number of spikes in each burst from pattern 2 to pattern 5 increased the ATP cost of neural activity at low *f*_intra_ (<150 Hz) but reduced the energy consumption at high *f*_intra_. With *f*_intra_ ≤75 Hz, the tonic patterns consumed less ATP than burst patterns. Note that the higher ATP cost required to reverse Na^+^ leakage at *f*_aver_ = 10 Hz arose from the long inter-burst intervals. At *f*_aver_ = 100 Hz, the ATP cost of burst patterns decreased as *f*_intra_ increased, and increasing number of spikes in each burst reduced the metabolic cost of neural activity (Fig. 4B). In this case, tonic patterns consumed more ATP than burst patterns.

**Figure 5:**
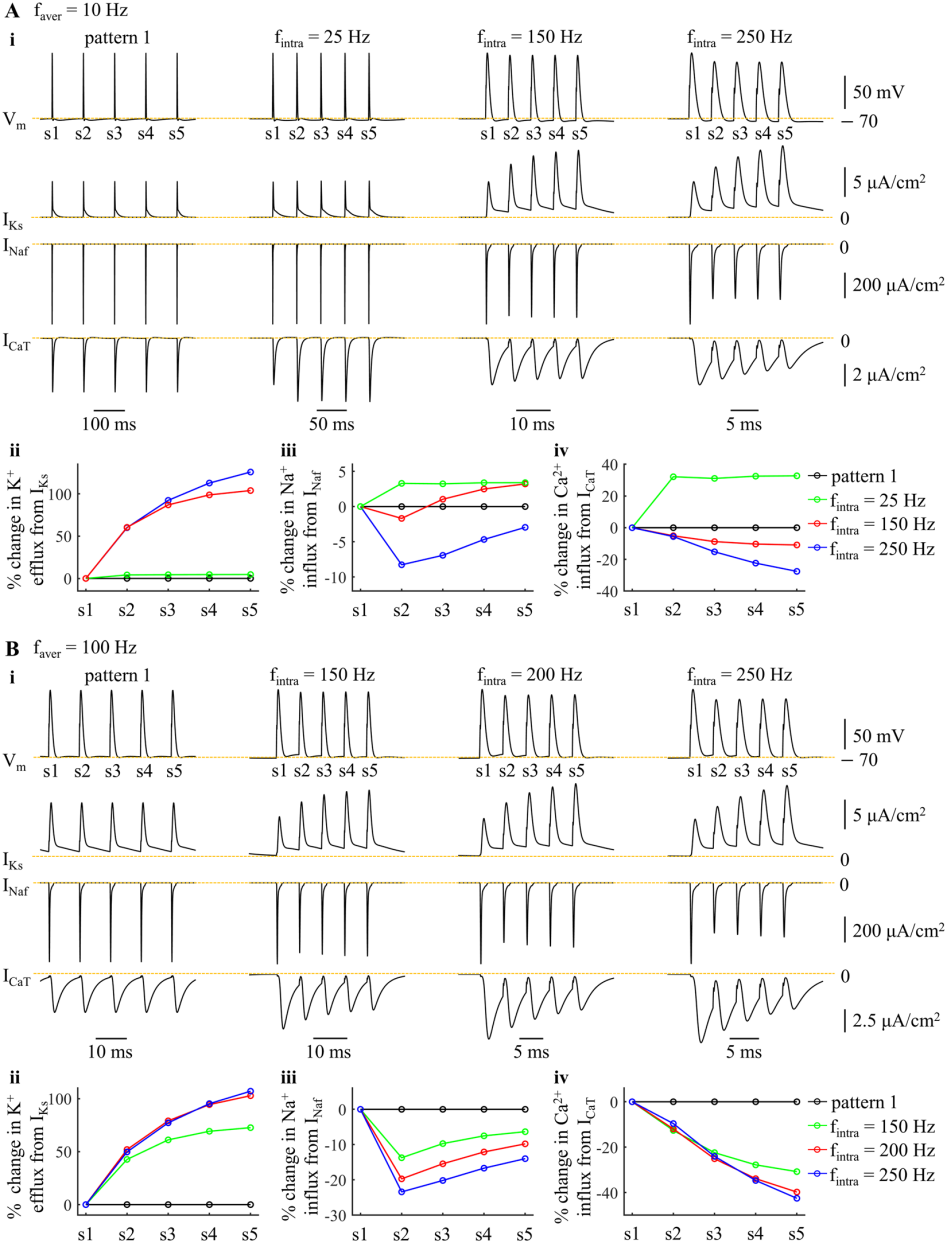
Effects of *f*_intra_ on ionic currents underlying patterns 1 and 5 at (**A**) *f*_aver_ = 10 Hz and (**B**) 100 Hz. (i) slow I_Ks_, fast I_Naf_, and low-threshold I_CaT_ underlying five APs. The mark numbers (i.e., s1–s5) of the spikes from each pattern were indicated in the top panels. For pattern 1 (left panels), I_Ks_, I_CaT_, and I_Naf_ were measured from 5 APs after 1000 ms. For pattern 5, currents were measured from a burst after 1000 ms. (ii) percent change in K^+^ efflux from I_Ks_ during each spike. (iii) percent change in Na^+^ influx from I_Naf_ during each spike. (iv) percent change in Ca^2+^ influx from I_CaT_ during each spike.

The I_Ks_, I_Naf_, and I_CaT_ in each burst showed distinct changes with *f*_intra_ (Fig. 5A,B, i). Increasing *f*_intra_ reduced the interspike intervals, and the activation of the kinetically-slow I_Ks_ built up from one spike to the next, especially at frequencies >100 Hz. This effectively increased the K^+^ efflux from I_Ks_ during each spike (Fig. 5A,B, ii). The changes in Na^+^ influx during each spike were the net effects of the afterpotentials and the augmented activation of I_Ks_ (Fig. 5A,B, iii). Due to the afterpotentials (Fig. 3B), the inward I_Naf_ in each burst was substantially reduced at high *f*_intra_, which resulted in a pronounced reduction in the Na^+^ load from APs. In contrast, the hyperpolarizing I_Ks_ overlapped with Na^+^ influx, and more Na^+^ influx was required to achieve AP depolarization after the 2^nd^ spike in each burst. Note that the changes in the Na^+^ influx of the 2^nd^ spike were dominated by the afterpotentials of the 1^st^ spike (Fig. 3B), since I_Ks_ was not sufficiently activated at this time. Unlike I_Ks_, the Ca^2+^ influx increased after the 2^nd^ spike in each burst at *f*_intra_ = 25 Hz, while it was reduced from one spike to the next at high *f*_intra_ (Fig. 5A,B, iv). Thus, the ATP expended on Ca^2+^ extrusion was higher at *f*_intra_ = 25 Hz than for tonic patterns, and then decreased with *f*_intra_ (Fig. 4C,D, center).

**Figure 6:**
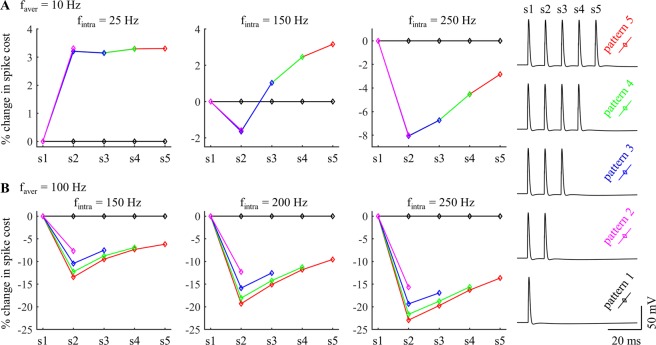
Effects of *f*_intra_ on percent change in spike cost of five patterns at (**A**) *f*_aver_ = 10 Hz and (**B**) 100 Hz. The APs were recorded from one period of each pattern after 1000 ms, and the mark numbers (i.e., s1–s5) of the APs are indicated in the right panel.

With *f*_aver_ = 10 Hz, the inter-burst interval was long enough that the afterpotentials of the last spike of the prior burst had little effect on the metabolic demand of the subsequent burst (Fig. 6A). At low *f*_intra_, the afterpotentials of the cell body and the augmented activation of I_Ks_ both increased the Na^+^ influx during the APs (Fig. 6A, *f*_intra_ = 25 Hz), and, as well, the ATP consumed on extruding Ca^2+^ entry was increased from pattern 1 to pattern 5 (Fig. 5A, iv), resulting in higher metabolic costs of burst patterns than tonic patterns. Similarly, increasing the number of spikes in each burst increased the ATP cost of neural activity. At moderate *f*_intra_, the afterpotentials reduced the Na^+^ influx during each AP, but the augmented I_Ks_ of patterns 3–5 dominated the effects of afterpotentials after the 3^rd^ AP in each burst, resulting in a higher metabolic cost per spike than in tonic patterns (Fig. 6A, *f*_intra_ = 150 Hz). At high *f*_intra_, the effects of afterpotentials overpowered the effects of slow I_Ks_, and the ATP cost per spike in each burst were all lower than that of tonic patterns (Fig. 6A, *f*_intra_ = 250 Hz). Since the ATP cost from I_CaT_ was reduced from pattern 1 to pattern 5 at *f*_intra_ ≥100 Hz (Fig. 4C, center), tonic patterns required more ATP than burst patterns and increasing spike number in each burst reduced the metabolic cost of neural activity at high *f*_intra_. With *f*_aver_ = 100 Hz, the prior burst produced effects on the metabolic demand of subsequent bursts (Fig. 6B). Due to short interspike intervals in each burst, the reduction in I_Naf_ from the afterpotentials substantially reduced the AP metabolic cost, which dominated the effects of augmented I_Ks_, and burst patterns required less ATP than tonic patterns. Thus, the effects of varying *f*_intra_ on ATP consumption of neural activity arose from the interactions of these two currents and the different timescales of their kinetics.

### Metabolic cost with rebound activation

Burst and tonic modes differ in more than just firing pattern, and include differences in resting potential and spike threshold. By explicitly representing I_CaT_ in the model, we accounted for the effects of these other changes on the metabolic costs of neural activity. Hyperpolarization de-inactivates inward I_CaT_ in TC neurons and subsequent depolarization generates a low-threshold Ca^2+^ spike, which depends on the resting potential. The low-threshold spikes bring the membrane potential to the threshold for generating fast Na^+^ spikes, and result in a rebound burst of APs. We examined the effect of *f*_aver_ on the metabolic cost of firing patterns with rebound excitation generated by applying hyperpolarizing pulses to the cell body.

**Figure 7:**
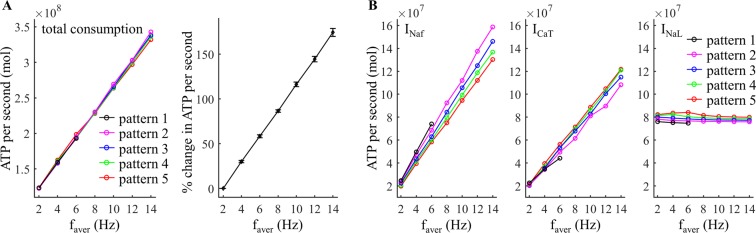
Effect of *f*_aver_ on estimated metabolic cost of different temporal patterns of neural activity resulting from rebound activation. (**A**) Left: total ATP consumption by neural activity. Right: percent change in total ATP consumption (mean ± SD across five patterns). (**B**) ATP consumption required to reverse the ionic fluxes through each of I_Naf_, I_CaT_, and I_NaL_. With *f*_aver_ ≥8 Hz, the interspike interval was so short that I_CaT_ was unable to steadily generate 1 spike during each rebound activation. Thus, the *f*_aver_ of pattern 1 was only from 2 Hz to 6 Hz.

**Figure 8:**
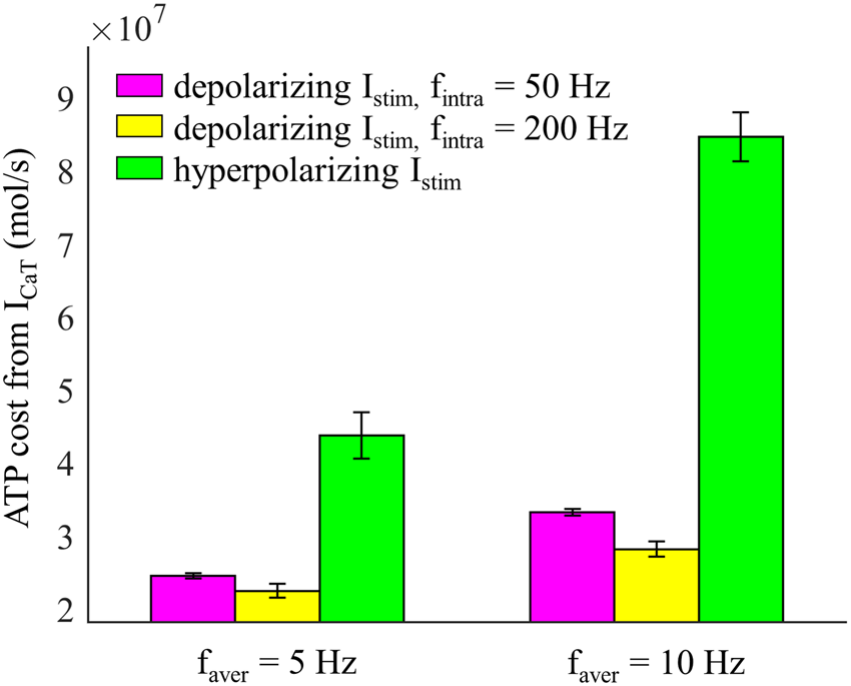
ATP cost per second of Ca^2+^ extrusion (mean ± SD). At *f*_aver_ = 5 Hz, the ATP cost was averaged over five firing patterns. At *f*_aver_ = 10 Hz, the ATP cost was averaged over patterns 2–5. With depolarizing I_stim_, inward I_CaT_ was inactivated and no rebound spikes occurred.

Increasing *f*_aver_ increased the number of APs, and the ATP cost of each pattern increased as a function of *f*_aver_ (Fig. 7A). A 2 Hz increment in *f*_aver_ increased the mean ATP cost across five patterns by ~29% (Fig. 7A, right), which was higher than ATP demand without rebound excitation (Fig. 2A,B, right). The increased number of spikes increased the ATP cost from fast Na^+^ and T-type Ca^2+^ currents, but did not alter appreciably the energy demand resulting from the Na^+^ leakage channel (Fig. 7B). When I_CaT_ was de-inactivated by hyperpolarization, Ca^2+^ extrusion consumed much more ATP than without rebound excitation (Fig. 8) and made an appreciable contribution to overall ATP demand.

**Figure 9:**
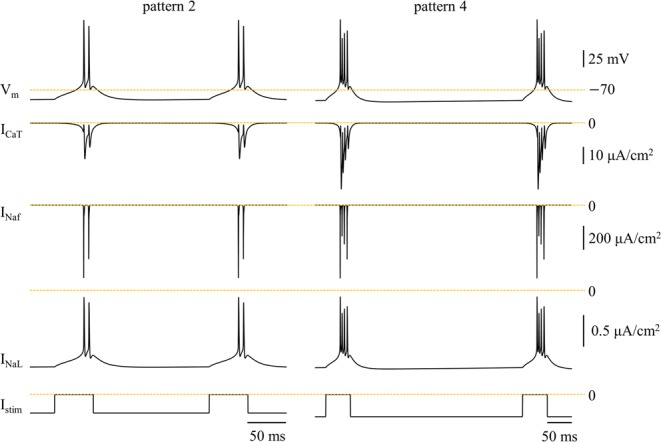
Ionic currents during rebound bursts at *f*_aver_ = 10 Hz. V_m_, I_CaT_, I_Naf_, and I_NaL_ were measured from firing patterns 2 (left) and 4 (right). Hyperpolarizing pulse train I_stim_ was applied to the cell body. The amplitude of the pulses was respectively −0.2 nA (left) and −0.24 nA (right).

The *f*_intra_ was high (>150 Hz) during rebound bursts following de-inactivation of I_CaT_, and with the same *f*_aver_, increasing numbers of spikes in each burst resulted in more APs falling within afterpotentials (Fig. 9). This reduced I_Naf_, and thus the ATP cost from the fast Na^+^ current. In contrast, stronger hyperpolarization to increase the number of spikes in each burst increased I_CaT_, which required more ATP for reversal. Stronger hyperpolarization also slightly increased I_NaL_, which resulted in a higher ATP cost. As a result, there was little difference in the total energy consumption between firing patterns, similar to when activity was driven by depolarizing current pulses.

## Discussion

We used a biophysically-based computational model of a TC relay neuron to estimate the metabolic cost of different temporal patterns of neural activity. We applied current pulses to the cell body to generate the tonic and burst modes of firing and quantified the effects of average firing rate, intraburst frequency, and spike number in each burst on ATP demand. The average firing rate was the dominant factor in determining the metabolic cost of neural activity, and the temporal pattern of activity contributed substantially less to determining the ATP required to maintain homeostasis of ion concentrations within the cell.

Estimates of energy requirements of neural computation indicated that APs made a significant contribution to the overall energy demand^10,11^. To restore ion concentrations, the Na^+^/K^+^ pump extrudes Na^+^ ions and imports K^+^ ions against their electrochemical gradients, thus consuming ATP. The energy demand from APs in different cell types and species was determined in earlier studies^12–15,23^, and it was predicted that ATP consumption was dependent on the firing rate^13–17,24^. The stimulus applied in these studies was a constant current, which not only altered the firing rates but also itself affected the AP cost. Unlike these prior studies, we focused on the metabolic costs of different temporal patterns of neural activity. The applied stimuli were brief current pulses, which themselves produced little effect on the energy cost of an evoked spike while controlling the rate and pattern of neural activity. Using a computational model, we showed that the average firing rate dominated the ATP consumption in both tonic and burst patterns of firing. Our simulations were consistent with experimental recordings^18,19^, which suggested that the metabolic demand of neural activity in the lateral superior olive, hippocampal CA1 region, and cerebral cortex exhibited a strong dependence on firing rate.

Earlier studies predicted that temperature^13,24^, cell size^15^, spike shape^12^, AP threshold^25^, channel densities and kinetics^12,14^, spike-frequency adaptation^17^, and dendritic properties^16^ influenced the energy cost of an AP by altering the underlying ionic currents. We extended these results to show that the afterpotentials in the cell body affected the activation of voltage-dependent Na^+^ currents and thus Na^+^ influx and spike metabolic cost. Further, biophysical models^16,17^ predicted that slow currents interact with the fast Na^+^ current to modulate the AP cost in a spike train, including the Ca^2+^-activated K^+^ (I_AHP_) current, voltage-activated K^+^ (I_M_) current, and dendritic high-voltage activated Ca^2+^ current. Our simulations indicated that varying the intraburst frequency altered the slow K^+^ and T-type Ca^2+^ currents underlying the APs in each burst, and these currents interacted with fast Na^+^ currents to contribute to the small differences in metabolic cost between firing patterns. These findings highlight the importance of slow ionic currents in determining the spike pattern-related energy demand in the brain.

The Ca^2+^ influx via T-type Ca^2+^ channels is a key regulator of cellular excitability^26–28^ and controls the switch between tonic and burst firing modes in TC relay neurons^1^. Further, intracellular Ca^2+^ signaling powerfully regulates the metabolism in multiple subcellular process^27,29^. The Ca^2+^ ATPase and the Na^+^-Ca^2+^ exchanger both participate in the control of neuronal Ca^2+^ signaling^11^. The former consumes one ATP for extruding one Ca^2+^ ion and predominates at low Ca^2+^ load. The Na^+^-Ca^2+^ exchanger uses the energy stored in the Na^+^ gradient to drive Ca^2+^ extrusion, which predominates at higher Ca^2+^ loads. It exports one Ca^2+^ ion in exchange for the import of three Na^+^ ions, which requires additional ATP for the extrusion of extra Na^+^ by the Na^+^/K^+^ pump. Our simulations showed that Ca^2+^ extrusion made a significant contribution to the metabolic cost of neural activity when the T-type Ca^2+^ current was de-inactivated. Intracellular Ca^2+^ signaling also participates in ATP production in mitochondria^27^, and we did not consider this in our simulations.

Quantifying the metabolic demand of neural activity influences the interpretation of functional brain imaging data through related metabolic mechanisms. Deep brain stimulation (DBS) is a neurosurgical method successful in treating movement disorders and alters the temporal patterns of neural activity^30–32^. fMRI is based on the changes in local circulation and metabolism^33^, and is used to measure and interpret changes in brain activity during DBS. fMRI studies of thalamic DBS reported that the evoked BOLD responses were sensitive to the stimulus frequency^34–36^. Our studies showed that the average firing rate dominated the metabolic cost of neural activity in TC relay neurons, suggesting that metabolism-dependent functional imaging methods may be sensitive to stimulation frequency-dependent changes in neural activity. *In vivo* experiments also indicate that the temporal pattern of DBS is a factor determining its effectiveness^37–39^, but we did not observe the significant effects of firing pattern on ATP demand. However, this does not necessarily mean the temporal patterns may not differentially affect the BOLD signal. It is possible that the burst and tonic modes of firing evoke different neurovascular coupling mechanisms and lead to distinct blood flow responses, which would be apparent in fMRI.

There were several limitations of our modeling approach. First, due to the limited data available on the magnitude and location of all the voltage-gated channels underlying the neural activity in TC cells, there may be over- or under-estimates of absolute metabolic costs, but this should not impact our comparisons of relative metabolic demands across firing rates and patterns. Second, we only examined the metabolic cost of firing patterns in the cell body. There are substantial differences in the energy efficiency of APs in the dendrites, soma, axon initial segment, and nodes of Ranvier^40^, and these differences may influence the overall metabolic cost of different patterns of firing. Third, we only considered the metabolic cost of tonic and burst patterns in a TC relay neuron model. These firing patterns may also be generated in other cell types, and their ionic currents and morphologies are substantially different than TC relay neurons, thus influencing the metabolic cost of firing activity. Fourth, the synaptic conductances were set such that when we used synaptic inputs to generate the desired firing patterns each presynaptic event evoked a spike in the soma. In our simulations there were only 165 excitatory synaptic inputs on the dendrites, and this represented only a small fraction of the total synapses on TC relay neurons, estimated to be 5584–8797, with ~65% being non-GABAergic^41,42^. Thus, the individual synaptic conductance for each modeled synapse was larger than that measured for single synapses on thalamic neurons, estimated to be 9–15 pS^43,44^. Finally, we did not include synaptic transmission in metabolic cost calculation, and this could influence overall ATP consumption. Pre- and post-synaptic mechanisms mediating synaptic transmission made a significant contribution to the overall usage of signaling-related energy in neurons and glia^10,45^.

In summary, we quantified the metabolic cost of different rates and patterns of activity in a model TC relay cell. The average firing rate determined the ATP demand of neural activity by directly determining the number of APs. The temporal pattern contributed to the metabolic energy by altering the interactions of ionic currents on multiple timescales, but made a much smaller contribution than average firing rate to the overall energy demand. These predictions are important for understanding information processing in TC relay neurons and critical for interpreting the signals from metabolism-dependent modalities of functional brain imaging (e.g., fMRI).

## Supplementary information

Supplementary Information
