# Supplementary material for: Average firing rate rather than temporal pattern determines metabolic cost of activity in thalamocortical relay neurons
Source: Sci Rep. 2019 May 6;9:6940. doi: 10.1038/s41598-019-43460-8 (PMC6502890; doi:10.1038/s41598-019-43460-8)
Supplement: Supplementary file 1 — Supplementary Information [file 41598_2019_43460_MOESM1_ESM.docx]

Supplementary Information

**Average firing rate rather than temporal pattern determines metabolic cost of activity in thalamocortical relay neurons**

Guosheng Yi^1,5^, and Warren M. Grill^1,2,3,4*^

**Affiliation:**

1. Department of Biomedical Engineering, Duke University, Durham, NC, United States

2. Department of Electrical and Computer Engineering, Duke University, Durham, NC, United States

3. Department of Neurobiology, Duke University School of Medicine, Durham, NC, United States

4. Department of Neurosurgery, Duke University School of Medicine, Durham, NC, United States

5. School of Electrical and Information Engineering, Tianjin University, Tianjin, China

*** Corresponding author:**

Warren M. Grill

E-mail: warren.grill@duke.edu

Phone: (919) 660-5276

Fax: (919) 684-4488

Address: Fitzpatrick CIEMAS 1427, Box 90281, 101 Science Drive, Durham, NC 27708-0281 USA


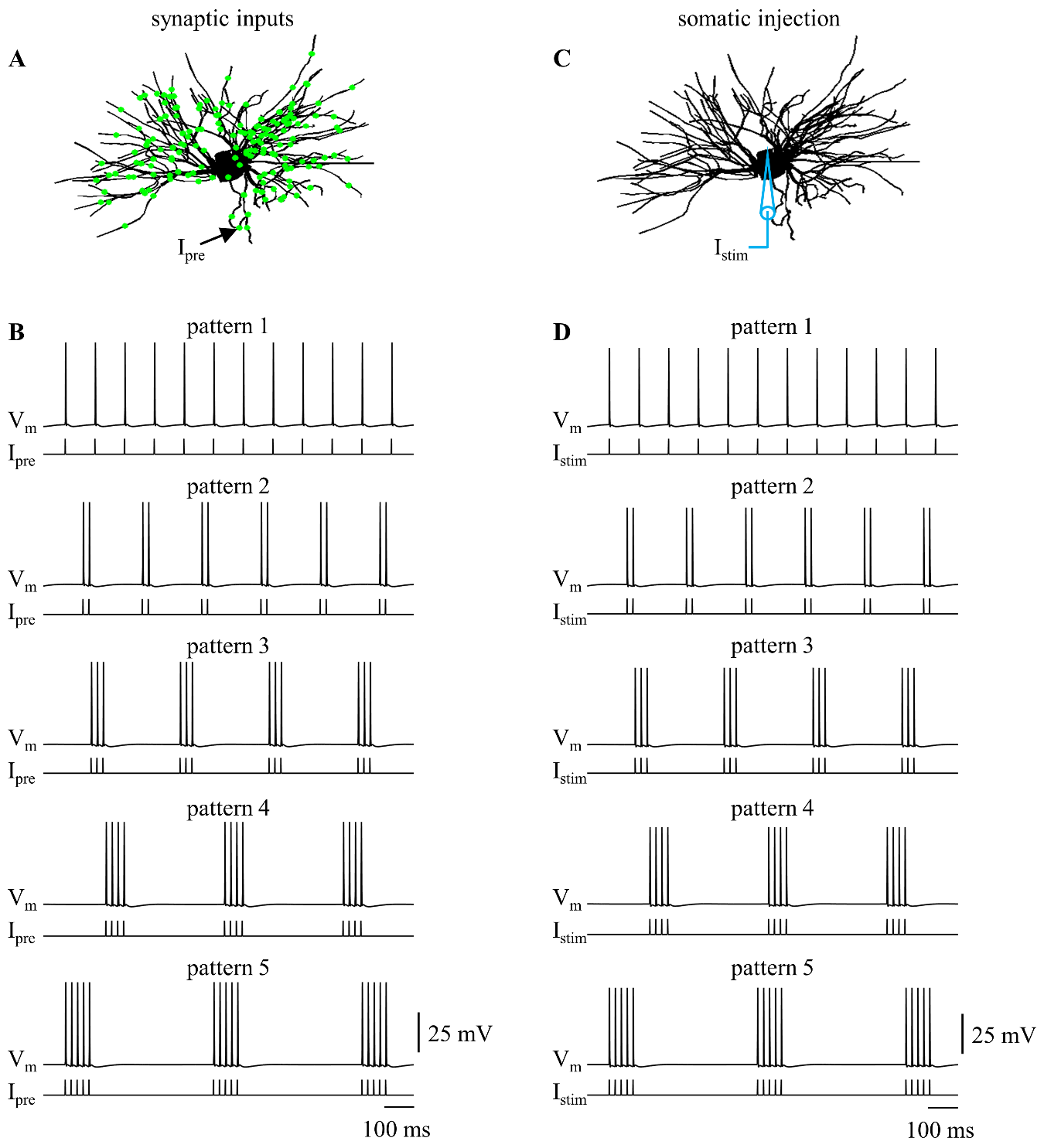


**Supplementary Figure S1.** **(A)** 165 excitatory synaptic inputs (green) were applied to the dendrites, which were modeled following our previous study [1]. Each synaptic input was activated by a presynaptic spike train I_pre_, and the patterns of presynaptic firing were set according to the desired patterns of somatic activity. The postsynaptic current generated by a presynaptic event was computed as $I_{\mathrm{syn}}\left( t \right)=g_{\mathrm{syn}}\frac{t}{\tau_{\mathrm{syn}}}e^{\left[ 1-t/{\tau_{\mathrm{syn}}} \right]}\left( V_{m}-E_{\mathrm{syn}} \right)$, where the reversal potential was *E*_syn_ = 0 mV and the time constant was *τ*_syn_ = 0.775 ms. **(B)** Neural activity generated by temporal pattern of excitatory synaptic inputs. Synaptic conductance was *g*_syn_ = 0.45 nS, which was suprathreshold for activation of a spike in the soma after each presynaptic event. **(C)** Depolarizing pulse train I_stim_ was applied to cell body. **(D)** Neural activity generated by somatic injection. Pulse width was 0.1 ms, and pulse amplitude was 40 nA.


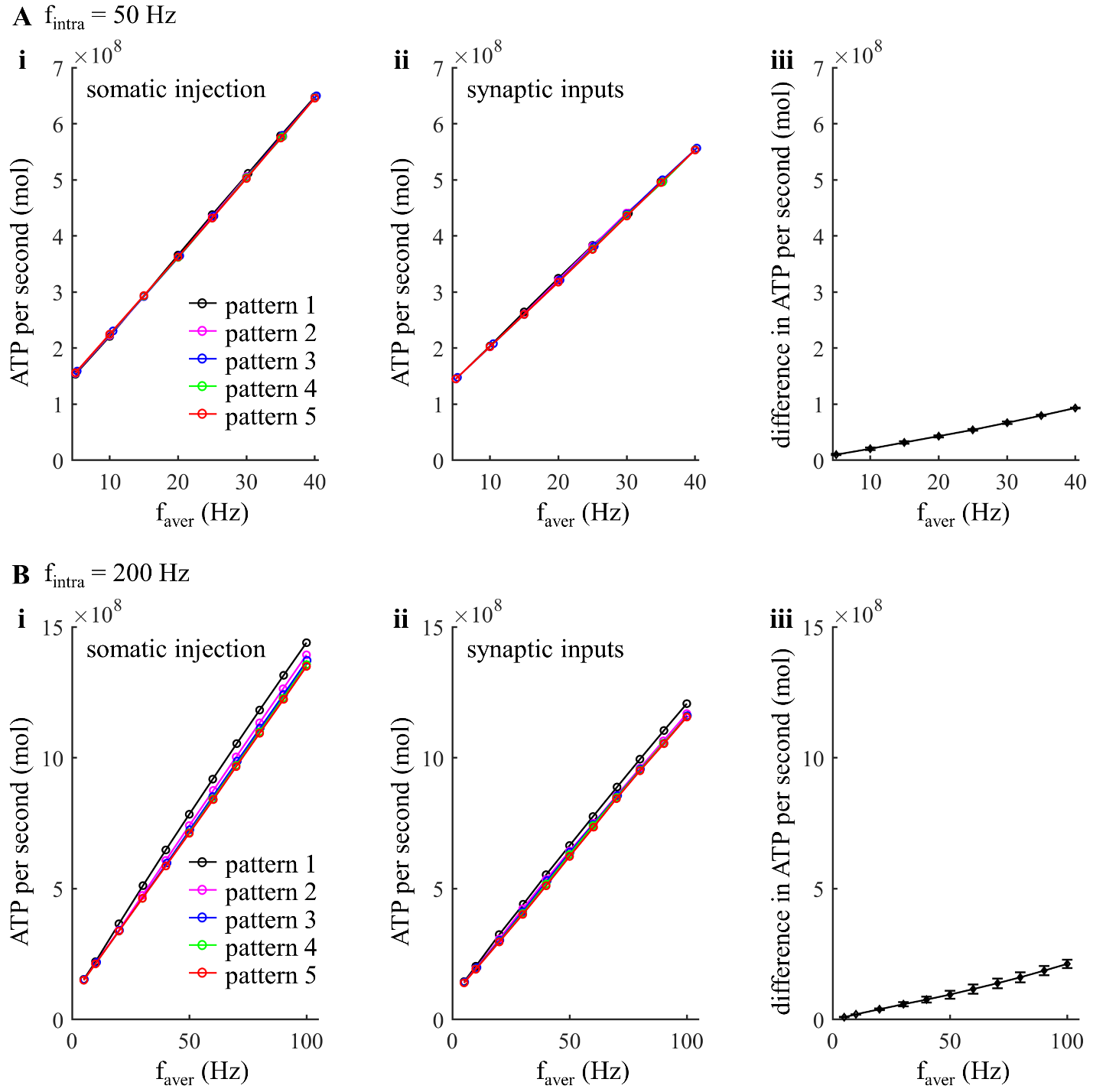


**Supplementary Figure S2.** Effect of average firing rate, *f*_aver_, on total ATP consumption by different patterns of neural activity at **(A)** *f*_intra_ $=$ 50 Hz and **(B)** 200 Hz. **(i)** Firing patterns were generated by somatic current injection. Pulse width was 0.1 ms and pulse amplitude was 40 nA. **(ii)** Firing patterns were generated by synaptic inputs. Synaptic conductance was *g*_syn_ = 0.45 nS. **(iii)** Difference between the ATP consumption in **(i)** and **(ii)** (mean ± SD across five patterns). Using somatic current injection and synaptic inputs distributed across the dendrites generated different somatic depolarizations, which resulted in the differences in ion fluxes and thus the ATP cost of the resulting firing patterns.


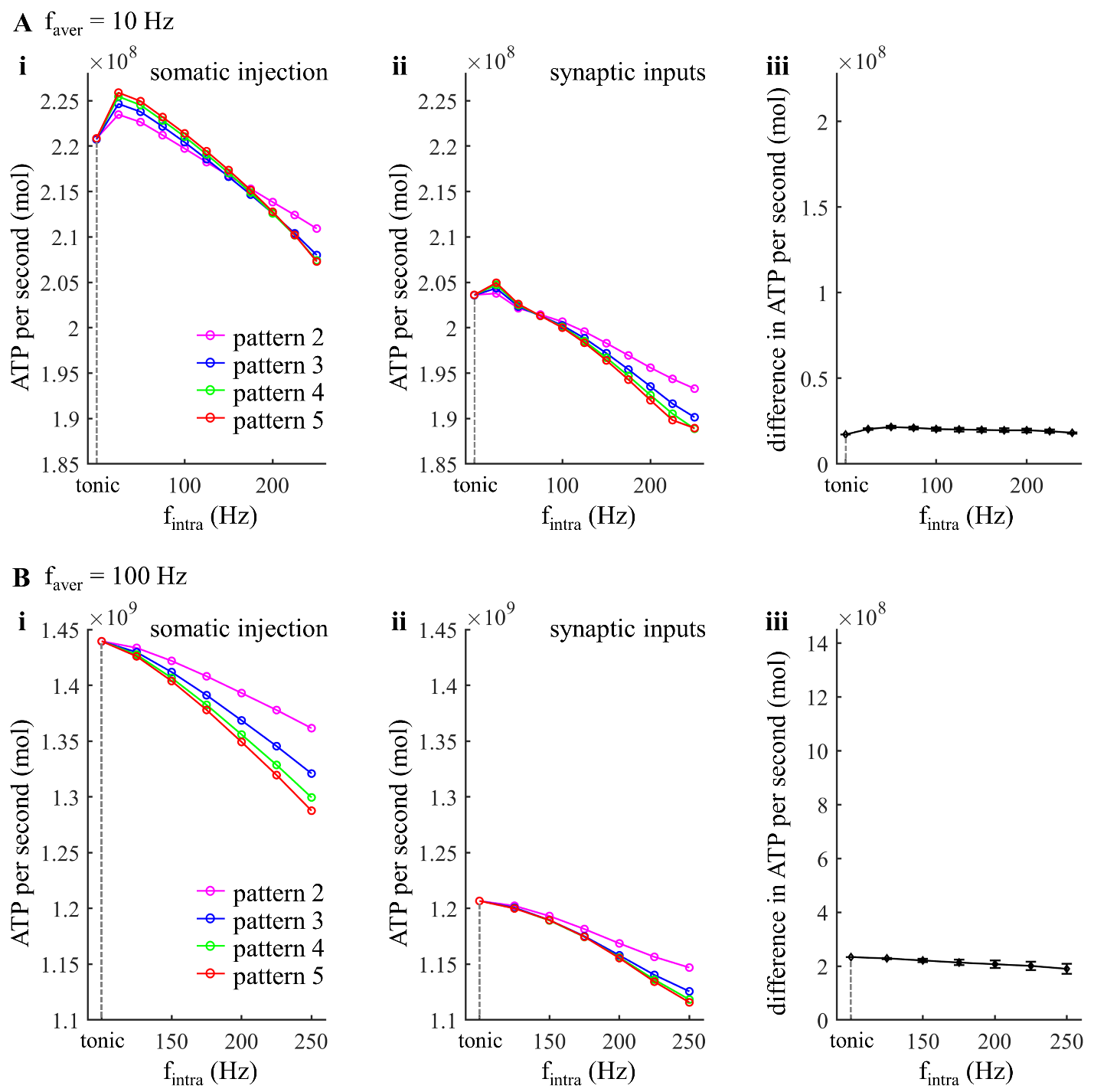


**Supplementary Figure S3.** Effect of intraburst frequency, *f*_intra_, on total ATP consumption of different patterns of neural activity at **(A)** *f*_aver_ $=$ 10 Hz and **(B)** 100 Hz. **(i)** Patterns of neural activity generated by somatic current injection. Pulse width was 0.1 ms and pulse amplitude was 40 nA. **(ii)** Patterns of neural activity generated by synaptic inputs. Synaptic conductance was *g*_syn_ = 0.45 nS. **(iii)** Difference between the ATP consumption in **(i)** and **(ii)** (mean ± SD across five patterns).

**Reference**

1. Yi, G. S., & Grill, W. M. Frequency-dependent antidromic activation in thalamocortical relay neurons: effects of synaptic inputs. *J. Neural Eng.* **15**, 056001 (2018).
